# Endometriosis of the Cervix: A Rare Clinical Case with the Possibility of Comparing the Eutopic and Ectopic Endometrium at the Cellular Level

**DOI:** 10.3390/ijms24032184

**Published:** 2023-01-22

**Authors:** Konstantin A. Toniyan, Elena Yu. Gorbacheva, Valery V. Boyarintsev, Irina V. Ogneva

**Affiliations:** 1 Gynecology Department, FGBU KB1 (Volynskaya) UDP RF, 121352 Moscow, Russia; 2 Cell Biophysics Lab., State Scientific Center of the Russian Federation Institute of Biomedical Problems of the Russian Academy of Sciences, 123007 Moscow, Russia; 3 Emergency and Extreme Medicine Department, FGBU DPO CGMA UDP RF, 121359 Moscow, Russia

**Keywords:** cervix, cytoskeleton, DNA methylation, endometrium, epigenetics, genital endometriosis

## Abstract

Endometriosis of the cervix is a rare form of genital endometriosis, which is characterized by the appearance of tissue on the vaginal part of the cervix, similar to the tissue of the mucous membrane of the uterine cavity. We describe a clinical case in which we compared the content of cytoskeletal proteins, H3 histone modifications and DNA methylation (total and 5-hydroxymethylcytosine content) in the eutopic endometrium and in tissue from endometriosis foci on the cervix. The patient had elevated levels of estradiol, interleukin-1β and interleukin-8. At the cellular level, the content of tubulin and the marker of stable microtubules were reduced in the ectopic endometrium (by 45% and 37%, *p* < 0.05, respectively), but the alpha-actinin-1 content was increased (by 75%, *p* < 0.05) with an increase in the expression of its gene. At the same time, the total level of DNA methylation in the endometriotic focus was reduced by more than 2 times with the accumulation of the intermediate product 5-hydroxymethylcytosine (the content increased by more than 3 times), probably due to an increase in the content of tet methylcytosine dioxygenase 1 (more than 4 times).

## 1. Introduction

To date, endometriosis, which is the appearance of ectopic foci of the endometrium, is one of the most common gynecological diseases. The formation of endometrioitic lesions is usually associated with changes in hormonal background and immunological status [1,2,3,4,5], more often with the provocative effect of trauma, for example, during childbirth or surgery [6]. However, even in the absence of such a stimulus, endometrial cells, nevertheless, may have an increased migration potential associated with a change in the content of cytoskeletal proteins and the expression of their genes [7,8].

The ectopic foci of endometriotic tissue can have various localizations, including extremely rare thoracic [9,10] and nasolacrimal [11,12] localization, but, most often, genital localization occurs. However, cases of the localization of haemorrhagic cystic lesions assuming a dark blue or chocolate brown appearance on the vaginal part of the cervix are quite rare.

Endometriosis of the cervix is one of the causes of recurrent metrorrhagia, including in the form of postcoital bleeding [6]. In rare cases, patients experience menorrhagia, but most often cervical endometriosis is asymptomatic and the pathology is detected by chance, for example, during colposcopy, biopsy/conization of the cervix or after hysterectomy [13].

Below, we describe a case of asymptomatic cervical endometriosis in a patient without previous traumatization of the cervix. The reason for the planned hospitalization was the need to remove the endometrial polyp with separate diagnostic curettage. The latter made it possible to compare the eutopic (from the uterine cavity) and the ectopic (from the surface of the cervix) endometrium at the cellular level.

## 2. Results

### 2.1. Clinical Case

A 46-year-old patient was admitted to the gynecological department with a referral diagnosis: an endometrial polyp for planned surgical treatment.

The patient was in a stable clinical condition with no clinical, microbiological or laboratory evidence of infection, encephalopathy, renal failure, comorbidities including heart failure or pulmonary disease, malignancy, diabetes mellitus. Written informed consent was obtained from the patient prior to participation in the study. The study design and all procedures were approved by the Biomedicine Ethics Committee of the Institute of Biomedical Problems, Russian Academy of Sciences (Physiology Section of the Russian Bioethics Committee, Russian Federation National Commission for UNESCO, Permit #523/MSK/09/26/19) and conformed to the Declaration of Helsinki.

Gynecological anamnesis: the duration of the menstrual cycle—28 days, regularly, the duration of menstruation—4–5 days, average, painful. There was one pregnancy that ended in operative delivery (caesarean section) 12 years previously; the patient denies other surgical interventions.

Hormonal status: FSH—13.56 mIU/mL, LH—66.27 mIU/mL, progesterone—0.81 ng/mL, estradiol—733.60 pg/mL *.

Immunological status: interleukin-1β and interleukin-8 levels were increased (Table 1).

**Figure 1:**
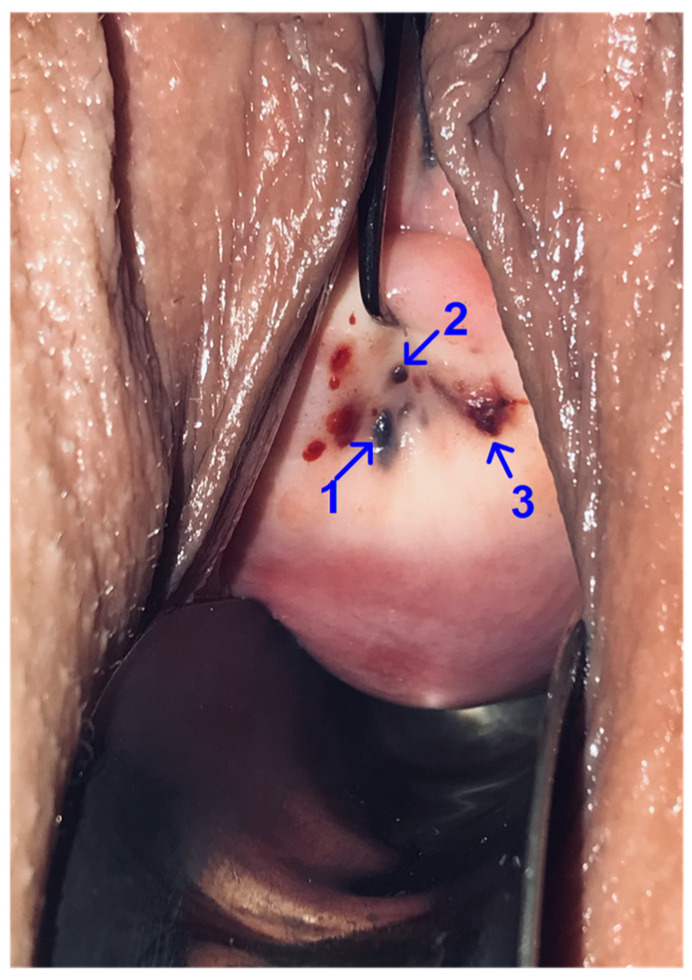
Localization of endometriotic lesions on the cervix during speculum exam. Foci of ectopic endometrium, up to 2 mm in size: 1—at 7 o’clock of the conditional dial, 2—in the area of the external os of the cervix, 3—the formation of a purple color at 5 o’clock of the conditional dial. The patient gave informed consent to the photographic recording and publication of the photograph.

On gynecological examination: the body of the uterus in anteflexio, not enlarged, dense, mobile, painless on palpation. Adnexa of the uterus on both sides is not defined. The vaginal discharge is light. Subsequent speculum exam revealed: the cervix is cylindrical, the external os of the cervix is in atresia; three dot formations up to 2 mm in size are determined on the cervix (Figure 1).

A polypectomy was performed by hysteroresectoscopy and separate diagnostic curettage, after which the biomaterial was sent for histological examination; no atypical cells were detected. Part of the eutopic endometrium from the uterine cavity was frozen in liquid nitrogen for further study.

Endometrial lesions were removed during cervical biopsy. Part of the biomaterial was sent for histological examination, confirming the presence of endometriosis. Another part of the ectopic endometrium was frozen in liquid nitrogen for examination.

### 2.2. The relative Content of Cytoskeletal Proteins, Expression of Its Genes and Epigenetic Events in Eutopic and Ectopic Endometrium

**Figure 2:**
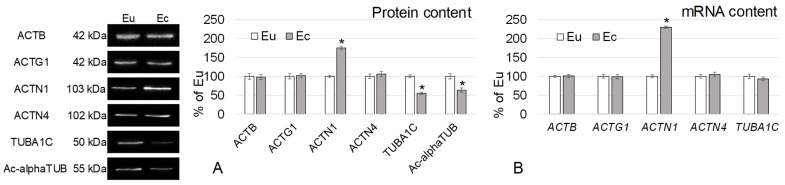
The relative cytoskeletal protein content (**A**) with Western-blots and mRNA content (**B**) in the eutopic and ectopic endometrium. Eu—eutopic endometrium. Ec—ectopic endometrium. ACTB—beta-actin. ACTG1—gamma-actin. ACTN1—alpha-actinin-1. ACTN4—alpha-actinin-4. TUBA1C—alpha-tubulin. Ac-alphaTUB—acetylated alpha-tubulin. Western-blotting was performed twice, qRT-PCR—thrice. *—*p* < 0.05 in comparison with Eu.

The relative content of actin isoforms (beta- and gamma-), actin-binding protein alpha-actinin-4, as well as the mRNA of the corresponding genes, did not differ in biopsies of the eutopic and ectopic endometrium (Figure 2). The content of alpha-actinin-1 in the focus of endometriosis was 75% (*p* < 0.05) (Figure 2A) and the mRNA encoding its gene was higher by 130% (*p* < 0.05) (Figure 2B) than in the eutopic endometrium. The content of alpha-tubulin and acetylated alpha-tubulin was reduced in the ectopic endometrium by 45% (*p* < 0.05) and 37% (*p* < 0.05), respectively, although the mRNA level did not differ from the norm.

**Figure 3:**
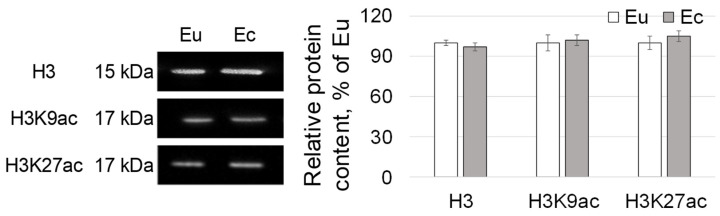
The relative protein content of the histone H3 and its acetylated modifications with Western-blots in the eutopic and ectopic endometrium. Eu—eutopic endometrium. Ec—ectopic endometrium. H3—histone H3. H3K9ac—histone H3 acetylLys10. H3K27ac—histone H3 acetylLys28. Western-blotting for H3 and H3K9ac was performed twice, for H3K27ac—thrice.

The relative content of histone H3, as well as its isoforms acetylated at 9 and 27 lysine (H3K9ac and H3K27ac) did not differ in the biopsy specimens of the eutopic and ectopic endometrium (Figure 3).

**Figure 4:**
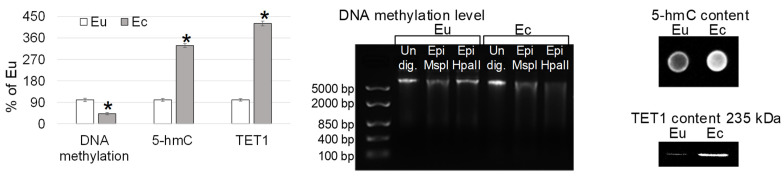
The epigenetic events in the eutopic and ectopic endometrium: total DNA methylation level, the relative content of 5-hmC and active demethylase TET1 (tet methylcytosine dioxygenase 1) with typical pictures. Eu—eutopic endometrium. Ec—ectopic endometrium. Undig.—undigested DNA. Epi MspI—digested with Epi MspI. Epi HpaII—digested with Epi HpaII. Western-blotting and restriction analysis were performed twice, dot-blotting—thrice. *—*p* < 0.05 in comparison with norm Eu.

In the ectopic endometrium, the level of total DNA methylation was lower than in the eutopic endometrium by 58% (*p* < 0.05) (Figure 4). At the same time, the main changes in the level of methylation were associated with the methylation of internal cytosine at the 5′-CCGG-3′ locus, since no changes in the efficiency of MspI (which is blocked by the methylation of external cytosine) were observed during the treatment of cells of the eutopic endometrium and endometrial focus. The relative contents of 5-hydroxymethylcytosine (5-hmC) and active demethylase TET1 in the cells of the endometrial focus were higher by 228% (*p* < 0.05) and 321% (*p* < 0.05), respectively, than in the normal endometrium (Figure 4).

## 3. Discussion

The formation of an ectopic endometrial focus is possible only with the combined action of several factors: on the one hand, there must be a change in the structure of the cell layer in the place where the focus arises so that the endometrium-like cell can attach and begin to proliferate, against the background of changes in the immune status; on the other hand, endometrial cells must undergo some changes, in particular, in the ability to migrate, adhere and subsequently proliferate in an unusual environment.

In the described clinical case, the patient had changes in immunological status that are characteristic of endomteriosis: an increase in the blood levels of interleukin-1β and interleukin-8 (Table 1). It is generally accepted that IL-1β stimulates the cyclooxygenase system 2, leading to the growth of the ectopic endometrium by inducing proliferation and angiogenesis as a result of an increase in the production of growth factor VEGF [14,15,16,17], and the latter may also be mediated by IL-8 [18,19].

In addition, the patient had a significantly increased level of estradiol, which regulates the remodeling of the actin cytoskeleton in endometrial cells, leading to an increase in their migration potential [20,21]. Therefore, we analyzed the content of the cytoskeletal proteins, the expression of genes encoding them and their regulation in biopsies of the eutopic endometrium (obtained by separate diagnostic curettage) and the ectopic endometrium (from foci located on the cervix).

The results obtained indicated that the content of actin isoforms did not change, as well as the actin-binding protein alpha-actinin-4. However, the content of its homologue, alpha-actinin-1, increased, as did the expression of its gene (Figure 2). Alpha-actinin-1 is an actin-binding protein that, on the one hand, binds the cortical actin cytoskeleton to the membrane, and, on the other hand, ensures its interaction with cytoplasmic signaling proteins [22]. ACTN1 belongs to the spectrin family [23] and functions as an antiparallel homodimer, linking the ends of actin filaments to each other [24]. The binding of ACTN1 to actin is necessary for the formation of stress fibrils and cell adhesion/motility, but phosphorylation of the tyrosine residue by focal adhesion kinase, as well as an increase in intracellular calcium concentration above 10^−7^ M, completely inhibits this interaction [25,26]. In addition, mutations in the ACTN1 gene lead to a change in the shape of cells, in particular platelets, and a violation of their adhesive ability. In the human ACTN1 gene, six missense mutations (one in 1, 2, 3, 7 exon and two in 18 exon) associated with autosomal dominant congenital macrothrombocytopenia have been identified [27,28]. At the same time, the overexpression of the ACTN1 gene usually indicates an increase in the migration potential of cells [29,30,31], but the literature data are given primarily for malignant cells. Previously, we have already shown an increase in the content of ACTN1 in endometriosis of various localizations, increasingly significant the farther from the eutopic localization the focus of ectopic endometrium was located [8].

The malignancy of the focus of endometriosis is an extremely rare phenomenon [32,33], which may be due to a decrease in the content of tubulin, which forms microtubules, which we noted in extragenital forms of endometriosis [8]. Even for cancers, a favorable prognostic criterion is a decrease in microtubule formation [34,35]. For this case of genital endometriosis, it can be assumed that a decrease in the content of stable microtubules (their marker is acetylated tubulin, the content of which is reduced) and tubulin monomers (Figure 2A) reduces the efficiency of fission spindle formation and, accordingly, prevents the pronounced proliferation of endometrial cells in the ectopic focus.

Thus, the protein pattern of endometrial cells localized in the ectopic site compared to the eutopic site indicates a decrease in stable microtubules and an increase in the content of actin-binding protein with an increase in the expression of genes encoding them.

Changes in gene expression in mammals, in particular in humans, can be associated with various modifications of chromatin (for example, changes in the level of histone acetylation) and DNA (for example, methylation and changes in the content of the intermediate product 5-hydroxymethylcytosine). In the described clinical case, the content of histone H3 isoforms acetylated at lysines 9 and 27 did not differ in the cells of the eutopic and ectopic endometrium (Figure 3). At the same time, in the ectopic endometrium, a decrease in the total level of DNA methylation was observed with the accumulation of the intermediate product 5hmC, possibly due to an increase in the content of TET1 demethylase (Figure 4), which actively demethylates the genome [36,37,38]. This pattern of DNA methylation is usually associated with an increase in gene expression [39], which may be the reason for the increase in *ACTN1* gene expression in the ectopic endometrium in this clinical case. Moreover, interestingly, when comparing the endometrium without evidence of atypia and endometrial carcinoma, TET1 and 5-hmC levels were significantly higher in the normal endometrium [40]. Accordingly, the accumulation of 5-hmC and an increase in the content of TET1 in the ectopic endometrium can be considered as a favorable prognostic sign in assessing the possible risk of the malignancy of the endometriotic lesion.

## 4. Methods

### 4.1. Western Blotting for the Evaluation of Relative Protein Content

Frozen tissues were used for protein extraction. Samples were homogenized in Laemmli buffer containing a protease inhibitor cocktail (Calbiochem, San Diego, CA, USA) on ice. The protein concentration in each sample was measured and, accordingly, the same amount of protein was applied to the wells of the polyacrylamide gel. After denaturing electrophoresis, proteins were transferred onto nitrocellulose membranes followed by staining with specific primary antibodies (Table 2) and their corresponding HRP-conjugated secondary antibodies (anti-rabbit #7074S, an-ti-mouse #7076S, all Cell Signaling Technology, Danvers, MA, USA). Next, membranes were treated with substrates (SuperSignal™ West Femto Maximum Sensitivity Substrate, Thermo Scientific, Waltham, MA, USA), detected using ChemiDoc XRS+ imaging system (Bio-Rad Laboratories, Hercules, CA, USA) and processed using Image Lab Software (Bio-Rad Laboratories, Hercules, CA, USA).

### 4.2. RT-PCR for the Evaluation of the Relative mRNA Level

Total RNA from frozen tissues was isolated using an RNeasy Micro Kit (#74004, Qiagen, Hilden, Germany) according to the manufacturer’s instructions. Reverse transcription was performed using d(T)_15_ as a primer with 500 ng of RNA. qPCR was performed using the Mx300P system (Stratagene, La Jolla, CA, USA) using SYBR green with specific primers (Table 3). The expression of target genes was normalized to histone H3 and quantified by the 2^−ΔΔCT^ method.

### 4.3. Restriction Analysis (MspI/HpaII) for the Determination of DNA Total Methylation Level

To determine the methylation levels, total DNA was isolated from the frozen tissues using a DNA extraction kit (Syntol, Moscow, Russia) based on the phenol/chloroform method according to the manufacturer’s instructions. For the total CpG methylation analysis at the 5′-CCGG-3′ locus, an EpiJET Methylation Analysis Kit (MspI/HpaII) (Thermo Scientific, Waltham, MA, USA) was used according to the manufacturer’s instructions. The restriction results analysis was carried out in a 1% agarose gel with a size marker, FastRuler Middle Range DNA Ladder (Thermo Scientific, Waltham, MA, USA), and the results were processed using Image Lab Software (Bio-Rad Laboratories, Hercules, CA, USA), with normalization of the total content of the undigested genomic DNA in the corresponding sample.

### 4.4. Dot Blot Method for the Determination of the 5-Hydroxymethylcytosine (5hmC) Content in DNA

The 100 ng isolated DNA (described in the previous section) was applied to a nitrocellulose membrane, for the preliminarily measurement of the concentration and for denaturing (+95 °C for 5 min and then +4 °C for 3 min) at three technical replicas. The membranes were air-dried, welded to the membrane using ultraviolet light and incubated in 4% skim milk overnight at +4 °C. To evaluate the 5hmC content, we used specific primary antibodies (#ab214728, 1 μg/mL, Abcam, Cambridge, UK) and then processed membranes as described above (in the Western blotting section).

### 4.5. Statistical Analysis

Each method was performed at least twice with consistent results. The Mann–Whitney U-test was used to assess difference between groups. Data were presented as Mean ± SEM (for technical replicas). A value of *p* < 0.05 was considered statistically significant.

## 5. Conclusions

The localization of the endometrioid focus on the cervix is quite rare and the debut of this disease is usually associated with trauma, for example, in natural childbirth, abortion or destructive manipulations. In the described clinical case, there was no history of such trauma and the detection of endometrioitic lesions was an incidental finding during a planned colposcopy before polypectomy. However, the patient showed a change in immunological status, which contributed to the survival of the ectopic endometrium. In addition, an increased level of estradiol could lead to a change in the content of cytoskeletal proteins as a result of changes in the expression of the corresponding genes against the background of an aberrant pattern of DNA methylation. Moreover, such a cellular profile contributes to an increase in the migration potential of cells. Thus, in this clinical case, there was a combined effect of all three leading factors in the development of endometriosis: an aberrant immunological status, hormonal levels and changes in cell structure, but the patient did not have any clinical manifestations of the disease.

The limitations of the study are due to its type—a clinical case. The required replicas from molecular studies were technical. A positive aspect of the study is associated with the possibility of comparing the eutopic and ectopic endometrium in the same patient, which may provide ideas for finding new approaches to the prediction, diagnosis and treatment of endometriosis.

## Figures and Tables

**Table 1:** Levels of cytokines in a female suffering from cervix endometriosis.

Cytokines	Result, pg/mL	Reference, pg/mL
IL-1β	7.08 *	<5.00
IL-2	1.8	<10
IL-4	1.06	<25
IL-6	5.4	<7
IL-8	66.3 *	<62.0
IL-10	2.6	<9.1
IL-12	3.14	<10
IL-18	205.766	104–650
IFNα	0.91	<5
IFNγ	7.32	<189
TNFα	3.99	<6

*—increase relative to the reference value.

**Table 2:** Primary antibodies.

Protein	Manufacturer with Catalog Number, Dilution
ACTB (beta-actin, 42 kDa)	Santa Cruz Biotechnology, Inc., Santa Cruz, CA, USA, #sc-81178, 1:300
ACTG1 (gamma-actin, 42 kDa)	Santa Cruz Biotechnology, Inc., Santa Cruz, CA, USA, #sc-65638, 1:100
ACTN1 (alpha-actinin1, 103 kDa)	Santa Cruz Biotechnology, Inc., Santa Cruz, CA, USA, #sc-17829, 1:500
ACTN4 (alpha-actinin4, 102 kDa)	Santa Cruz Biotechnology, Inc., Santa Cruz, CA, USA, #sc-393495, 1:100
TUBA1C (alpha-tubulin, 50 kDa)	Abcam, Cambridge, UK, #ab52866, 1:1000–1:50,000
Ac-alphaTUB (acetylated alpha-tubulin, 55 kDa)	Santa Cruz Biotechnology, Inc., USA, #sc-23950, 1:300
H3 (histone H3, 15 kDa)	Abcam, Cambridge, UK, #ab10799, 1 mkg/mL
H3K9ac (histone H3 acetylLys10, 17 kDa)	Abcam, Cambridge, UK, #ab4441, 1:10,000
H3K27ac (histone H3 acetylLys28, 17 kDa)	Abcam, Cambridge, UK, #ab4729, 1 mkg/mL
TET1 (tet methylcytosine dioxygenase 1, 235 kDa)	Abcam, Cambridge, UK, #ab191698, 2 mkg/mL

**Table 3:** Primer sequences and product sizes.

Gene	Primer Sequence, Forward/Reverse (5′…3′)	Product Size, bp
*ACTB*	CTCGCCTTTGCCGATCC/TCTCCATGTCGTCCCAGTTG	298
*ACTG1*	GTTTCTCTGCCGGTCGCAAT/CCGACGATGGAAGGAAACA	126
*ACTN1*	GTGTCCGCCTAGTTCAGTGT/ATTGACCGCCAACACTTTGC	251
*ACTN4*	AATCCAATGAGCACCTCCGC/TGGTGTGCTTGTTGTCGAAG	243
*TUBA1C*	CCGGCCACCCTTTCACTACT/CTCATCGTCTCCTTCAGCACT	76
*H3F3A*	AATCGACCGGTGGTAAAGCA/GACGCTGGAAGGGAAGTTTG	183

## Data Availability

All data generated or analyzed during this study are included in this article.
